# Mapsnp: An R Package to Plot a Genomic Map for Single Nucleotide Polymorphisms

**DOI:** 10.1371/journal.pone.0123609

**Published:** 2015-04-08

**Authors:** Fuquan Zhang, Yong Xu, Hongbao Cao, Chunhui Jin, Zaohuo Cheng, Guoqiang Wang, Yin Yao Shugart

**Affiliations:** 1 Wuxi Mental Health Center of Nanjing Medical University, Wuxi, Jiangsu Province, China; 2 Unit on Statistical Genomics, Intramural Research Program, National Institute of Mental Health, National Institutes of Health, Bethesda, Maryland, United States of America; 3 Department of Psychiatry, First Hospital of Shanxi Medical University, Taiyuan, China; Children's Medical Research Institute, AUSTRALIA

## Abstract

Single-nucleotide polymorphism (SNP) is one of the most common sources of genetic variations of the genome. Currently, SNPs are a main target for most genetic association studies. Visualizing genomic coordinates of SNPs, including their physical location relative to their host gene, and the structure of the relevant transcripts, may provide intuitive supplements to the understanding of their functions. Nevertheless, to date, no such easy-to-use programming tools exist. Therefore, we developed an R package, “mapsnp”, to plot genomic map for a panel of SNPs within a genome region of interest, including the relative chromosome location and the transcripts in the region. mapsnp is a simple and flexible software package which can be used to visualize a genomic map for SNPs, integrating a chromosome ideogram, genomic coordinates, SNP locations and SNP labels.

## Introduction

Single-nucleotide polymorphism (SNP) is one of the most common sources of genetic variations of the genome. Currently, many genetic studies, including genome-wide association study (GWAS) use SNPs as their research tools. Visualizing SNP data in a graph may provide a clear and intuitive impression for the reader. Various visualization tools have been developed, most of which have been implemented in the form of a genome browser. The NCBI genome browsers [1] distributes genome annotations and provides integrated views of valuable genomic data for supported genomes. The UCSC [2] and Ensembl Genome Browser [3] offer online tools for visualizing a set of annotation ‘tracks’ for a genomic region. These web applications provide analysis and retrieval resources and serve as reference datasets for individual SNPs or genes. Nevertheless, these tools have limited plotting options and are not programmatically accessible. For example, these tools cannot be used to yield annotation tracks for a specific set of SNPs within a genomic region of interest, due to their lack of flexibility.

The statistical programming environment R [4] provides a plethora of methods and tools to analyze and visualize data. There are several programming tools for visualizing genomic data, such as GenomeGraphs [5], ggbio [6], and Gviz [7]. Within these packages, individual types of genomic features or data are represented by separate tracks, and there are constructor functions to coordinate and plot these tracks. Yet, none of these packages provide a specific function to plot detailed information for a panel of user-specified SNPs.

We developed mapsnp, as an add-on software package for the statistical programming environment R, to facilitate the integrated visualization of genomic maps for SNPs of interest.

## Methods and Implementations

### Program description

Our function leverages the statistical functionality available in R, the grammar of graphics and the data handling capabilities of the Bioconductor project [8]. mapsnp employs the Gviz system [7] to plot a genomic map for candidate SNPs. Biological scientists often confront the need to plot genomic data, along with a variety of genomic annotation features, such as gene or transcript models, CpG island, and genetic variations. These features may be extracted from public data bases like PubMed, Ensembl, or UCSC. The Gviz package provides a structured visualization framework to plot any type of data along genomic coordinates. All plots are done using the grid graphics system, and several specialized annotation classes allow the integration of publicly available genomic annotation data from sources like UCSC [2] or Ensembl [3]. The rationale behind the Gviz package is that individual types of genomic features or data are represented by separate tracks. Each track constitutes a single object inheriting from class GdObject, and there are constructor functions to plot these tracks. When combining multiple objects, the individual tracks will always share the same genomic coordinate system, thus automatically aligning the plot elements.

A SNP map plotted by mapsnp includes five tracks: a chromosome ideogram track, a genomic coordinate track, a transcript track, a SNP location track, and a SNP label track.

An ideogram is a simplified visual representation of a chromosome, with the different chromosomal staining bands indicated by color, and the centromere indicated by shape. The necessary information to produce this visualization is stored in online data repositories, for instance at UCSC (http://genome.ucsc.edu/). Unlike all other track objects, ideogram tracks are not displayed on the same coordinate system. Instead, the current genomic location is indicated on the chromosome by a red box that sits on the ideogram image. This track shows the currently displayed region in the broader context of the whole chromosome, indicating how much of the complete chromosome is covered in our plot.

The genomic coordinate track is always relative to the other plotted tracks, thus there is little need for additional function arguments to adjust its output.

For the transcript track, the ‘msa’ function calls the biomaRt package [9] to perform on-line annotation queries to Ensembl [3] and then translates these to transcript structures. The ‘msb’ function utilizes Homo Sapiens data from UCSC based on the knownGene table, implemented in the ‘TxDb.Hsapiens.UCSC.hg19.knownGene’ package [10].

The SNP location track indicates genomic base-pair location of SNPs, while the SNP label track annotates their ID number.

Since the mapsnp package inherits from the Gviz package, detailed rationales or algorithms for each of these tracks can be found at Gviz’s online user guide (http://www.bioconductor.org/packages/release/bioc/html/Gviz.html).

### Functionality

The mapsnp package contains two functions, ‘msa’ and ‘msb’. Both functions have three necessary arguments, ‘M’, ‘start’, and ‘end’. The major parameter ‘M’ is a three-column matrix or data frame, including chromosome, SNP ID, and SNP’s genomic location. The ‘start’ and ‘end’ indicate the range of a highlighting region, typically a gene region corresponding to the mapped SNPs. The package offers multiple options to fine-tune the track appearance. Display parameters control the look of a track by defining properties of individual track objects.

The genomic coordinate track determines plotted genomic range. There is no need to know in advance about a particular genomic location when constructing the map. Instead, the plotted genomic range will be determined automatically from the context. Our package will display the region from the leftmost item to the rightmost item in any of the tracks. We often wish to zoom in or out on a particular plotting region to see more details or to get a broader overview. To that end, the ‘start’ and ‘end’ arguments let us choose an arbitrary genomic range to plot. Another pair of arguments that control the zoom state are ‘extendL’ and ‘extendR’. These arguments are relative to the currently displayed ranges, and can be used to extend the view on one or both ends of the plot. The argument ‘axisLabPos’ controls the arrangement of the tick marks. It takes one of the values ‘alternating’, ‘above’ or ‘below’.

For the transcript track, one can control the horizontal stacking of overlapping items using the ‘stacking’ display parameter. Presently, the three values squish (making best use of the available space), dense (no stacking at all) and hide (not showing the track items at all) are supported.

The ‘SNPidPos’ parameter controls the vertical positioning of the SNP ID labels, with three values (‘alternating’, ‘above’ or ‘below’). The ‘alternating’ will construct two SNP label tracks, with one above the SNP location track and the other below the SNP location track.

There are dozens of other parameters, which fine-tune other track properties, such as color, size, track name, annotation text, and so on. The detailed usage of the package is described in Supporting Information S1 File.

### Built-in SNP dataset

The built-in dataset involving seven candidate SNPs within the ATXN2 gene, from a genetic association study of schizophrenia [11]. As shown in Table 1, the dataset includes 7 SNPs within the ATXN2 gene, involving three columns: the first column is chromosome, the second column is SNP ID, and the third column is SNP genomic location.

**Table 1:** Seven SNPs in ATXN2

Chr	SNP_id	bp
12	rs2301621	111895272
12	rs6490162	111941120
12	rs630512	111952167
12	rs607316	111969448
12	rs616668	111974280
12	rs7969300	111993712
12	rs653178	112007756

Chr: chromosome; bp: base pair

## Results

### Application on the SNP data

To illustrate the use of mapsnp, we show an example for ‘msa’ on the built-in dataset involving seven candidate SNPs within the ATXN2 gene. The genomic range of this gene is from 111950277 to 112036294 base-pair.

Load the mapsnp package and the snp data:

- library(mapsnp)
- data(snp)
- msa(M = snp, start = 111950277, end = 112036294, SNPidPos = ‘alternating’)

**Fig 1:**
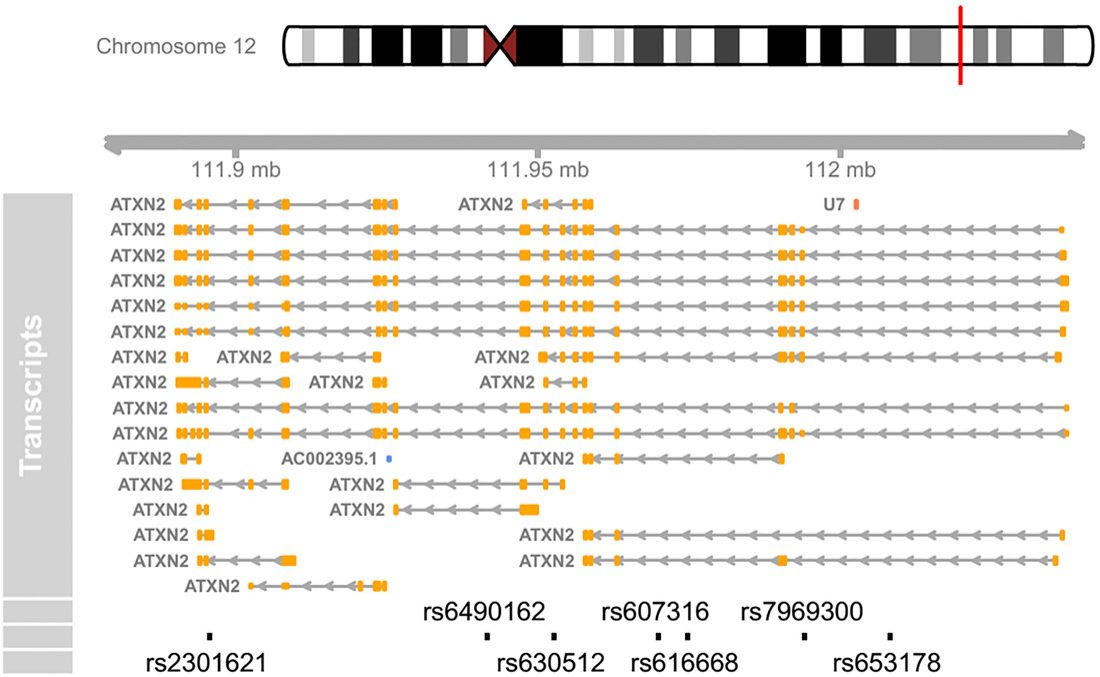
A detailed genomic map for seven SNPs within ATXN2 using Ensembl database. At the top, the relevant chromosome is drawn with the subregion of interest marked in red. The ‘Transcript’ track shows all the alternative transcripts within the region, retrieved from Ensembl database, including a non-ATXN2 transcript U7. At the bottom, the SNPs’ location and ID are plotted along the same genomic coordinate.

The above command plots a genomic map for the ATXN2 SNPs, with multiple alternative transcripts (Fig 1). Please note that users need an established internet connection for the package to work, and that fetching data from UCSC can take some time. Usually, there might be dozens of transcripts for a gene, and the longer a gene is, the more transcripts it has. Therefore, the transcript track may seem redundant for large genes. In many situations, it may be more desirable to create a concise map with all the transcripts stacking together. Nevertheless, there is a caveat in doing so, as there are some transcripts for other overlapping genes within the genomic region, for instance U7 and AC230095.1 in Fig 1. When combining multiple transcripts together, the intended transcript ATXN2 will contain other ‘noisy’ transcripts as well.

**Fig 2:**
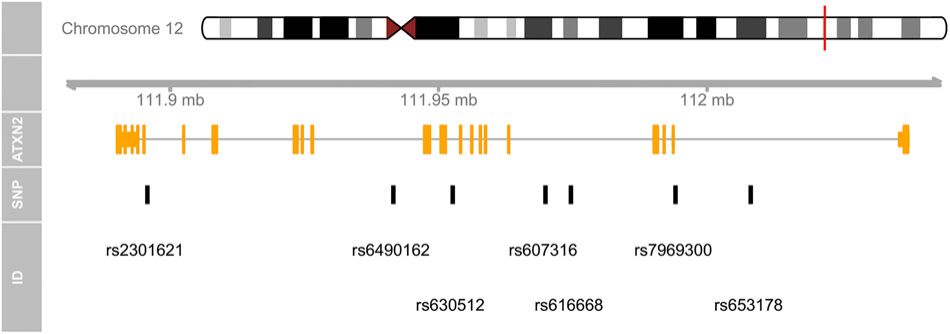
A concise genomic map for seven SNPs within ATXN2 using UCSC database. At the top, the relevant chromosome is drawn with the subregion of interest marked in red. The ‘ATXN2’ track shows the combined gene model of the alternative transcripts of the ATXN2 gene. At the bottom, the SNPs’ location and ID are plotted along the same genomic coordinate. > library(TxDb.Hsapiens.UCSC.hg19.knownGene) > msb(M = snp, start = 111950277, end = 112036294, geneTrName = ‘ATXN2’).

To circumvent the above issue, we created another function, msb, which leverages existent UCSC dataset curated by the ‘TxDb.Hsapiens.UCSC.hg19.knownGene’ package for the construction of a transcript track. It contains only the major transcripts within the genomic region. Thus, multiple transcripts can be safely merged together to produce a concise genomic map. The following commands will load the dataset and plot a genomic map for the ATXN2 SNPs, with only one merged transcript (Fig 2).

### Comparison with existing tools

Various visualization tools have been developed, most of which are implemented in the form of a genome browser. There are desktop-based browsers like Integrated Genome Browser [12] and Integrative Genomics Viewer [13], as well as web-based genome browsers like Ensembl, UCSC Genome Browser, and GBrowse [14]. These browsers can display genomic annotations and other features for a region, each with specific advantages for different purposes. However, web-based genome browsers cannot integrate custom-supplied data into the genomic map. Desktop-based browsers can display custom data alongside gene annotations, but they have difficulty manipulating feature tracks from foundation datasets and lack programming flexibility. Programming tools like GenomeGraphs [5], ggbio [6], and Gviz [7] have the flexibility to coordinate and plot public genomic features and custom data. However, none of these packages offer a specific function to show genomic information for a panel of candidate SNPs.

**Fig 3:**
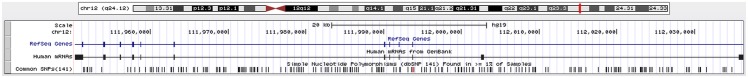
A genomic map for ATXN2 via UCSC browser.

**Fig 4:**
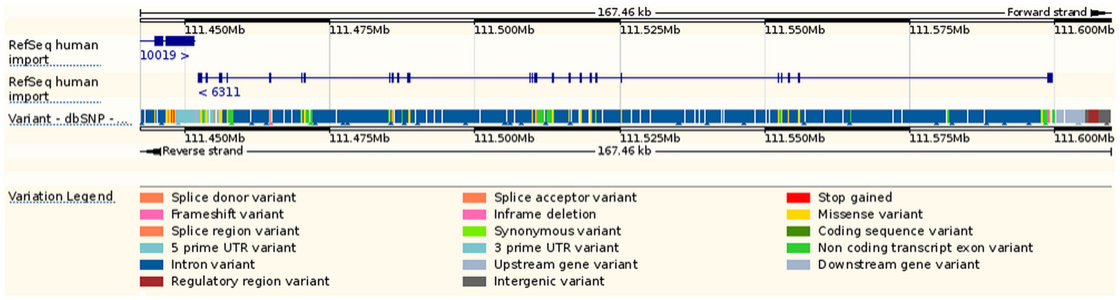
A genomic map for ATXN2 via Ensembl browser.

Since UCSC Genome Browser and Ensembl are two of the most commonly used genome visualization tools, we utilized these two browsers to illustrate the genomic information of ATXN2 gene. With UCSC browser, we input the ‘ATXN2’ and selected a subset tracks to show its genomic features (Fig 3). Next, we made a gene-based display for ATXN2 in Ensembl (Fig 4). As shown in Fig 3 and Fig 4, all of the common SNPs within ATXN2 were displayed. We could not select or highlight SNPs of interest within the gene. Fig 4 also lacks an ideogram track.

To our knowledge, mapsnp is the only software package specifically designed to display genomic information for SNPs of interest.

## Discussion

Visualization is an important component of genomic analysis, because it facilitates exploration and discovery by revealing genomic patterns of variations [6]. Presently, most visualization tools are implemented in the form of a genome browser, which are unable to address user-supplied SNPs. mapsnp provides a new tool to visualize and explore genomics annotations for a group of SNPs. It mimics the layout of the popular UCSC Genome Browser, providing detailed views of SNP locations, genomic regions, summary views of splicing patterns, and genome-wide overviews with a karyogram. Within the views, one can easily discern whether a SNP is located in exon, intron, 3’ untranslated regions (UTR) or 5’UTR of a transcript, as well as get an overview of the distribution of a panel of SNPs relative to their host gene. The package is especially useful for most candidate gene studies by exploring genomic features for relevant SNPs.

The two functions implemented in the package are easy to use. We used this package to plot one gene in the example. However, the package can plot multiple adjacent genes simultaneously. Meanwhile, the functions are flexible by offering dozens of parameters to fine-tune a final graph output, which are detailed in the package documentation. Future versions of the package will include more flexibility in terms of plotting parameters.

The major limitation of the mapsnp package may be its relatively slow executing speed, since the connection to the online data (UCSC) can take a certain amount of time (usually several minutes) depending on usage and network traffic. The other limitation lies in that only a single chromosome can be active during a given plotting operation. Consequently, we cannot plot multiple chromosomes in a single call to the function. However, we can build our own composite plots using multiple consecutive calls. It is best to plot no more than two or three dozen SNPs within a map, since too many SNPs will make it difficult to discriminate them.

In conclusion, we present mapsnp, a software tool designed to visualize genomic maps for SNPs of interest, integrating chromosome ideogram, genomic coordinates, SNP locations and SNP labels.

### Availability and requirements

The mapsnp package has been developed for the free R statistical environment (http://www.r-project.org) and runs under the major operating systems. The functions in the mapsnp package are accompanied by documentation files and simple examples to facilitate its use.

**Project name:** mapsnp

**Project home page:** https://github.com/csuzfq/mapsnp_pkg

**Operating system(s):** Platform independent.

**Programming language:** R.

**Other requirements:** R 2.15, Gviz 1.2.1, TxDb.Hsapiens.UCSC.hg19.knownGene 2.8.0.

**License:** GPL (≥3)

## Supporting Information

S1 File Package mapsnp . A PDF document of mapsnp manual.
(PDF)
