# Supplementary material for: Mapsnp: An R Package to Plot a Genomic Map for Single Nucleotide Polymorphisms
Source: PLoS One. 2015 Apr 8;10(4):e0123609. doi: 10.1371/journal.pone.0123609 (PMC4390227; doi:10.1371/journal.pone.0123609)
Supplement: S1 File — . A PDF document of mapsnp manual. (PDF) [file pone.0123609.s001.pdf]

Package ‘mapsnp’

March 25, 2014

Type Package

Title Plot genomic map for single nucleotide polymorphism

Author Fuquan Zhang [aut, cre]

Maintainer Fuquan Zhang <zfq777@gmail.com>

Depends R (>= 2.15.0)

Imports Gviz, TxDb.Hsapiens.UCSC.hg19.knownGene

Description A package to plot genomic map of single
nucleotide polymorphisms (SNPs), including the a
chromosome ideogram, transcripts of the gene on which
the SNPs map to, the genomic location of SNPs, and their ID number.

License GPL (>= 3)

Encoding UTF-8

LazyLoad yes

Version 0.1

Date 2014-03-25

Collate 'mapsnp-package.R' 'msa.R' 'msb.R'

R topics documented:

mapsnp-package 2
msa 2
msb 4
snp 6

Index **8**

`mapsnp-package` *The mapsnp Package*

Description

Plot genomic map for single nucleotide polymorphism.

Details

Package:	mapsnp
Type:	Package
Version:	0.1
Date:	2014-03-25
Depends:	R (>= 2.15.0)
Encoding:	UTF-8
License:	GPL (>= 3)
LazyLoad:	yes

A package to plot genomic map of single nucleotide polymorphisms (SNPs), including the a chromosome ideogram, transcripts of the gene on which the SNPs map to, the genomic location of SNPs, and their ID number.

Author(s)

Fuquan Zhang

`msa` *Plot genomic map for SNP, with all transcripts.*

Description

A function to plot genomic map of SNP using Ensembl dataset retrieved by biomaRt. The trancript track includes all the trancripts within a highlighting range. An internet connection is needed during running of the function.

Usage

```
msa(M, start = start, end = end, extendL = 1,
  extendR = 1, cex = 0.6, showLab.chr = TRUE,
  axisLabPos = "alternating", littleTicks = FALSE,
  geneTrName = "Transcripts", showLab.geneTr = TRUE,
  stackHt.geneTr = 0.5, SNPbpTrName = "SNP",
  SNPbpLab = 1:nr, showLab.SNPbp = FALSE, SNPbpWd = 1,
  SNPbpFill = "black", stackHt.SNPbpTr = 0.5,
  SNPidTrName = "ID", showLab.SNPid = TRUE, SNPidWd = 1,
  SNPidPos = "below", stackHt.SNPidTr = 0.2,
  stacking = "squish", genome = "hg19",
  reverseStrand = FALSE, background.panel = "white",
  background.title = "grey", ...)
```

Arguments

- `axisLabPos` Character vector, one in "alternating", "revAlternating", "above" or "below". The vertical positioning of the axis labels. Default to "alternating".
- `cex` Numeric scalar. The overall font expansion factor for the axis annotation text. Default to 0.6.
- `background.panel` A character, background color of track panel. Default to "white".
- `background.title` A character, background color of track name. Default to "grey".
- `end` An integer scalar with the genomic end coordinates for the highlighting range.
- `extendL` Numeric scalar, extending the plotting range to the left by times of width relative the 1/10 of length of the genetic region. Default to 1.
- `extendR` Numeric scalar, extending the plotting range to the right by times of width relative the 1/10 of length of the genetic region. Default to 1.
- `geneTrName` Character scalar of the transcript track name used in the title panel when plotting. Default to "Transcripts".
- `genome` The genome on which the track ranges are defined. Usually this is a valid UCSC genome identifier. Default to "hg19".
- `littleTicks` littleTicks=FALSE: Logical scalar. Add more fine-grained tick marks. Default to FALSE.
- `M` A three-column matrix or data.fram. The first column is Chromosome, e.g. 1, 2, 3, ..., X, Y, the second column is SNP ID, and the third column is SNP genomic location.
- `reverseStrand` By default all tracks will be plotted in a 5' -> 3' direction. It sometimes can be useful to actually show the data relative to the opposite strand. To this end one can use the reverseStrand display parameter, which does just what its name suggests. Default to FALSE.
- `showLab.chr` Logical scalar. Control whether to plot the chromosome ideogram track item identifiers. Default to TRUE.
- `showLab.geneTr` Logical scalar. Control whether to plot the transcripts track item identifiers. Default to TRUE.
- `showLab.SNPbp` Logical scalar. Control whether to plot the SNP location track item identifiers. Default to FALSE.
- `showLab.SNPid` Logical scalar. Control whether to plot the SNP ID track item identifiers. Default to TRUE.
- `SNPbpFill` Character or integer scalar. The fill color for SNP location items. Default to "black".
- `SNPbpLab` item labels for SNP location. Default to series from 1 to number of rows.
- `SNPbpTrName` Character scalar of the SNP location track name used in the title panel when plotting. Default to "SNP".
- `SNPbpWd` Integer vectors, times of width relative the 1/140 of length of the genetic region for the SNP location track items. Default to 1.
- `SNPidPos` Character vector, one in "alternating", "above" or "below". The vertical positioning of the SNP ID labels. Default to "alternating".
- `SNPidTrName` Character scalar of the SNP ID track name used in the title panel when plotting. Default to "ID".

SNPidWd
: Integer vectors, times of width relative the 1/20 of length of the genetic region for the SNP ID track items. Default to 1.

stackHt.geneTr
: Numeric between 0 and 1. Controls the vertical size and spacing between stacked elements. The number defines the proportion of the total available space for the stack that is used to draw the transcripts items. Default to 0.5.

stackHt.SNPbpTr
: Numeric between 0 and 1. Controls the vertical size and spacing between stacked elements. The number defines the proportion of the total available space for the stack that is used to draw the SNP location items. Default to 0.5.

stackHt.SNPidTr
: Numeric between 0 and 1. Controls the vertical size and spacing between stacked elements. The number defines the proportion of the total available space for the stack that is used to draw the SNP ID items. Default to 0.2.

stacking
: Object of class "character", the stacking type of overlapping items on the final plot. One in c(hide, dense, squish, pack,full). Currently, only hide (do not show the track items at all), squish (make best use of the available space) and dense (no stacking at all) are implemented. Default to "squish".

start
: An integer scalar with the genomic start coordinates for the highlighting range.

...
: Additional items which will all be interpreted as further display parameters. See settings and the "Display Parameters" section below for details.

Details

A function to plot genomic map of SNP using biomaRt

Examples

```
## Not run:
data(snp)
msa(M=snp,start=111950277,end=112036294)

## End(Not run)
```

---

msb *Plot genomic map for SNP, with major transcripts.*

Description

A function to plot genomic map of SNP using dataset from R package "TxDb.Hsapiens.UCSC.hg19.knownGene". The trancript track includes only major trancripts within a highlighting range.

Usage

```
msb(M, start = start, end = end, extendL = 1,
  extendR = 1, cex = 0.6, showLab.chr = TRUE,
  axisLabPos = "alternating", littleTicks = FALSE,
  geneTrName = "Gene", showLab.geneTr = TRUE,
  stackHt.geneTr = 0.5, SNPbpTrName = "SNP",
  SNPbpLab = 1:nr, showLab.SNPbp = FALSE, SNPbpWd = 1,
  SNPbpFill = "black", stackHt.SNPbpTr = 0.5,
```

```
SNPidTrName = "ID", showLab.SNPid = TRUE, SNPidWd = 1,
SNPidPos = "below", stackHt.SNPidTr = 0.2,
stacking = "dense", genome = "hg19",
reverseStrand = FALSE, background.panel = "white",
background.title = "grey", ...)
```

Arguments

axisLabPos	Character vector, one in "alternating", "revAlternating", "above" or "below". The vertical positioning of the axis labels. Default to "alternating".
cex	Numeric scalar. The overall font expansion factor for the axis annotation text. Default to 0.6.
background.panel	A character, background color of track panel. Default to "white".
background.title	A character, background color of track name. Default to "grey".
end	An integer scalar with the genomic end coordinates for the highlighting range.
extendL	Numeric scalar, extending the plotting range to the left by times of width relative the 1/10 of length of the genetic region. Default to 1.
extendR	Numeric scalar, extending the plotting range to the right by times of width relative the 1/10 of length of the genetic region. Default to 1.
geneTrName	Character scalar of the transcript track name used in the title panel when plotting. Default to "Transcripts".
genome	The genome on which the track ranges are defined. Usually this is a valid UCSC genome identifier. Default to "hg19".
littleTicks	littleTicks=FALSE: Logical scalar. Add more fine-grained tick marks. Default to FALSE.
M	A three-column matrix or data.fram. The first column is Chromosome, e.g. 1, 2, 3, ..., X, Y, the second column is SNP ID, and the third column is SNP genomic location.
reverseStrand	By default all tracks will be plotted in a 5' -> 3' direction. It sometimes can be useful to actually show the data relative to the opposite strand. To this end one can use the reverseStrand display parameter, which does just what its name suggests. Default to FALSE.
showLab.chr	Logical scalar. Control whether to plot the chromosome ideogram track item identifiers. Default to TRUE.
showLab.geneTr	Logical scalar. Control whether to plot the transcripts track item identifiers. Default to TRUE.
showLab.SNPbp	Logical scalar. Control whether to plot the SNP location track item identifiers. Default to FALSE.
showLab.SNPid	Logical scalar. Control whether to plot the SNP ID track item identifiers. Default to TRUE.
SNPbpFill	Character or integer scalar. The fill color for SNP location items. Default to "black".
SNPbpLab	item labels for SNP location. Default to series from 1 to number of rows.
SNPbpTrName	Character scalar of the SNP location track name used in the title panel when plotting. Default to "SNP".

SNPbpWd	Integer vectors, times of width relative the 1/140 of length of the genetic region for the SNP location track items. Default to 1.
SNPidPos	Character vector, one in "alternating", "above" or "below". The vertical positioning of the SNP ID labels. Default to "alternating".
SNPidTrName	Character scalar of the SNP ID track name used in the title panel when plotting. Default to "ID".
SNPidWd	Integer vectors, times of width relative the 1/20 of length of the genetic region for the SNP ID track items. Default to 1.
stackHt.geneTr	Numeric between 0 and 1. Controls the vertical size and spacing between stacked elements. The number defines the proportion of the total available space for the stack that is used to draw the transcripts items. Default to 0.5.
stackHt.SNPbpTr	Numeric between 0 and 1. Controls the vertical size and spacing between stacked elements. The number defines the proportion of the total available space for the stack that is used to draw the SNP location items. Default to 0.5.
stackHt.SNPidTr	Numeric between 0 and 1. Controls the vertical size and spacing between stacked elements. The number defines the proportion of the total available space for the stack that is used to draw the SNP ID items. Default to 0.2.
stacking	Object of class "character", the stacking type of overlapping items on the final plot. One in c(hide, dense, squish, pack,full). Currently, only hide (do not show the track items at all), squish (make best use of the available space) and dense (no stacking at all) are implemented. Default to "dense".
start	An integer scalar with the genomic start coordinates for the highlighting range.
...	Additional items which will all be interpreted as further display parameters. See settings and the "Display Parameters" section below for details.

Details

A function to plot genomic map of SNP using dataset of UCSC.hg19

Examples

```
## Not run:
library(TxDb.Hsapiens.UCSC.hg19.knownGene)
data(snp)
msb(M=snp,start=111950277,end=112036294)

## End(Not run)
```

---

snp *An example dataset*

Description

SNP map information including chromosome, SNP ID, and SNP location.

Usage

```
data(snp)
```

Format

A data.frame containing genomic information for 7 SNPs in the ATXN2 gene.

Source

Association analysis of a functional variant in ATXN2 with schizophrenia. Neurosci Lett. 2014.

References

Zhang F, et al. Association analysis of a functional variant in ATXN2 with schizophrenia. Neurosci Lett. 2014; 562: 24-7.

Index

*Topic **datasets**
    snp, 6
*Topic **package**
    mapsnp-package, 2

mapsnp-package, 2
msa, 2
msb, 4

snp, 6
